# Characterization of Genetic Diversity and Linkage Disequilibrium of *ZmLOX4* and *ZmLOX5* Loci in Maize

**DOI:** 10.1371/journal.pone.0053973

**Published:** 2013-01-24

**Authors:** Gerald N. De La Fuente, Seth C. Murray, Thomas Isakeit, Yong-Soon Park, Yuanxin Yan, Marilyn L. Warburton, Michael V. Kolomiets

**Affiliations:** 1 Department of Soil and Crop Science, Texas A&M University, College Station, Texas, United States of America; 2 Department of Plant Pathology and Microbiology, Texas A&M University, College Station, Texas, United States of America; 3 Corn Host Plant Resistance Research Unit, United States Department of Agriculture-Agricultural Research Service, Mississippi State, Jackson, Mississippi, United States of America; CNR, Italy

## Abstract

Maize (*Zea mays* L.) lipoxygenases (*ZmLOXs*) are well recognized as important players in plant defense against pathogens, especially in cross kingdom lipid communication with pathogenic fungi. This study is among the first to investigate genetic diversity at important gene paralogs *ZmLOX4* and *ZmLOX5*. Sequencing of these genes in 400 diverse maize lines showed little genetic diversity and low linkage disequilibrium in the two genes. Importantly, we identified one inbred line in which *ZmLOX5* has a disrupted open reading frame, a line missing *ZmLOX5*, and five lines with a duplication of *ZmLOX5*. Tajima's D test suggests that both *ZmLOX4* and *ZmLOX5* have been under neutral selection. Further investigation of haplotype data revealed that within the *ZmLOX* family members only *ZmLOX12*, a monocot specific *ZmLOX*, showed strong linkage disequilibrium that extends further than expected in maize. Linkage disequilibrium patterns at these loci of interest are crucial for future candidate gene association mapping studies. *ZmLOX4* and *ZmLOX5* mutations and copy number variants are under further investigation for crop improvement.

## Introduction

Lipids and their oxidized derivatives, oxylipins, play a role in plant reaction to stress and plant-microbe interactions [1], [2]. Lipid mediated interactions between pathogens and plants have gained increased attention, as a disruption of plant-microbe communication could provide an avenue for resistance to diseases [3]. In maize (*Zea mays* L.), the grain crop with the highest worldwide production [4], infection by the fungus *Aspergillus flavus* and drought stress are two major sources of grain loss worldwide [5]. *A. flavus* produces the carcinogenic mycotoxin, aflatoxin, a potent and highly-regulated liver carcinogen that causes stunting and chronic illness, or immediate death at high levels in humans and animals worldwide [6]. While quantitative resistance to *A. flavus* has been identified and selected for in maize, no major genes for resistance have been identified and the problem is complex [7]. The regulation of mycotoxin production in fungi is partially mediated by genes belonging to the lipoxygenase (LOX) family [8]. LOX genes are found in plant, fungal and animal kingdoms, and LOX mediated cross-kingdom interactions are hypothesized to be involved in the susceptibility of plants to fungal invasion and subsequent mycotoxin production [2]. However, the specific molecular signals from plants or fungi that trigger mycotoxin production are poorly understood.

LOX genes are non-heme iron-containing dioxygenases that catalyze the oxygenation of polyunsaturated fatty acids (PUFAs) [9], which are further processed into an estimated 400 metabolites including the well-known hormone jasmonic acid (JA) and green leaf volatiles (GLVs) [10]. Both JAs and GLVs are important plant defense signals that regulate and coordinate plant defense to stress within the plant and between the plant and other plants or pathogens [11]–[15]. LOX genes have been shown to be conserved across plant and mammalian genomes [16] and are subdivided into two main functional groups; 9-LOXs and 13-LOXs depending on which carbon on the fatty acid chain is oxygenated. A total of 13 different maize LOXs (*ZmLOX*s) with varying functions, localization, and regulation within the plant have been reported [17].

Of the 13 *ZmLOX*s, *ZmLOX4* (GenBank accession: mRNA DQ335762, protein ABC59687) located on chromosome 1, and *ZmLOX5* (GenBank accession: mRNA DQ335763, protein ABC59688), located on chromosome 5, are the two most closely related paralogs, sharing 94% sequence identity; however, they share only 40–67% sequence identity with other *ZmLOX*s [18]. *ZmLOX4* and *ZmLOX5* are 9-LOXs and are segmentally duplicated genes. Other pairs of closest paralogs include tandemly duplicated *ZmLOX*1 and *ZmLOX*2 and segmentally duplicated genes, *ZmLOX7* and *ZmLOX8*, and *ZmLOX10* and *ZmLOX11*. Each pair member is suspected to have distinct functionality [19], and *ZmLOX4* and *ZmLOX5* have distinct organ-specific and stress-induced expression patterns, suggesting differential involvement in diverse physiological processes. *ZmLOX4* is expressed mainly in the roots and the shoot apical meristem while *ZmLOX5* is expressed predominantly in the above ground organs, especially the silks [18]. Localization and expression data support the hypothesis that *ZmLOX4* (expressed in the roots) is involved in drought tolerance, while *ZmLOX5* (expressed in the silks) affects aflatoxin resistance. A previous study reported a quantitative trait locus (QTL) affecting aflatoxin contamination in bin 5.02, where *ZmLOX5* also maps [20], and another QTL affecting aflatoxin contamination was discovered in the adjacent bin (5.03) [21].

Allelic diversity provides functional variation on which breeder's selection programs can act. This variation can sometimes be masked by epistatic interactions and alleles of large effect at other loci. When a gene of interest is identified, the natural variation at that gene can be screened by sequencing across diverse varieties, to identify new alleles that might provide improved functionality, and validate the effect of unique alleles in near isogenic lines [22]. Functional allelic diversity may include sequence changes, structural changes of the genome, varied levels of gene expression, and changes in epigenetics [23]. The very high polymorphism [24] and low linkage disequilibrium [25] found in diverse maize populations requires a large number of markers to cover the genome for association analysis or, alternatively, a candidate gene (a gene believed to play a role in a trait of interest) must be identified in which to test associations [26], [27].

The published maize reference sequence [28] and the Maize HapMap [26], (1.4 million single nucleotide polymorphisms (SNPs) collected on 27 diverse maize lines) are invaluable resources for studies of genetic diversity and association mapping. The Maize HapMap may be problematic when working with paralogs in the genome, as the short reads created using next generation sequencing techniques may not distinguish between two genes with high similarity (such as *ZmLOX4* and *ZmLOX5*). For this reason, terminator dye sequencing and standard protocols were used to investigate the genetic diversity at the *ZmLOX4* and *ZmLOX5* loci. The identified genetic polymorphisms in these loci will be essential for using them in candidate gene association mapping studies.

## Results

### The genic structure and polymorphism of *ZmLOX4* and *ZmLOX5*

**Figure 1:**
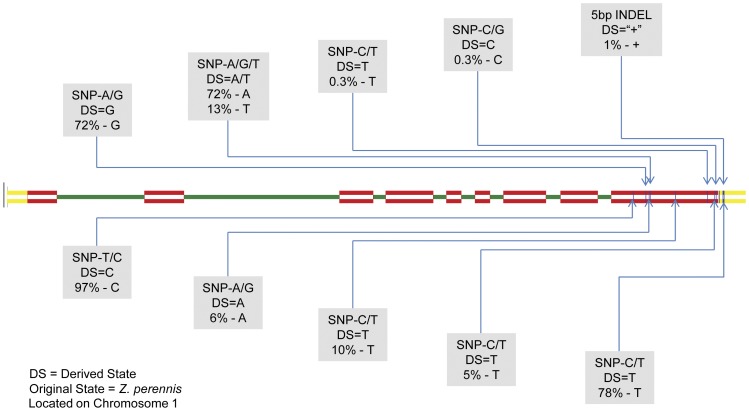
Sequence architecture of *ZmLOX4*. Similar to the *ZmLOX5* gene *ZmLOX4* consists of 9 exons (shown in red), and 8 introns (shown in green). The major difference between the architecture of the two genes is the length of the second intron, much larger in *ZmLOX4* spanning 11,191 bp (not shown). *ZmLOX4* is located on the forward strand of chromosome 1:264,209,651–264,226,078, spanning 16,427 bp (B73 RefGen_v2). Vertical blue lines in the final 3′ exon of the gene are the relative locations of SNPs discovered from Sanger sequencing. Their base pair change, derived state (as compared to *Z. perennis*), and derived state percentages are shown in the grey boxes.

**Figure 2:**
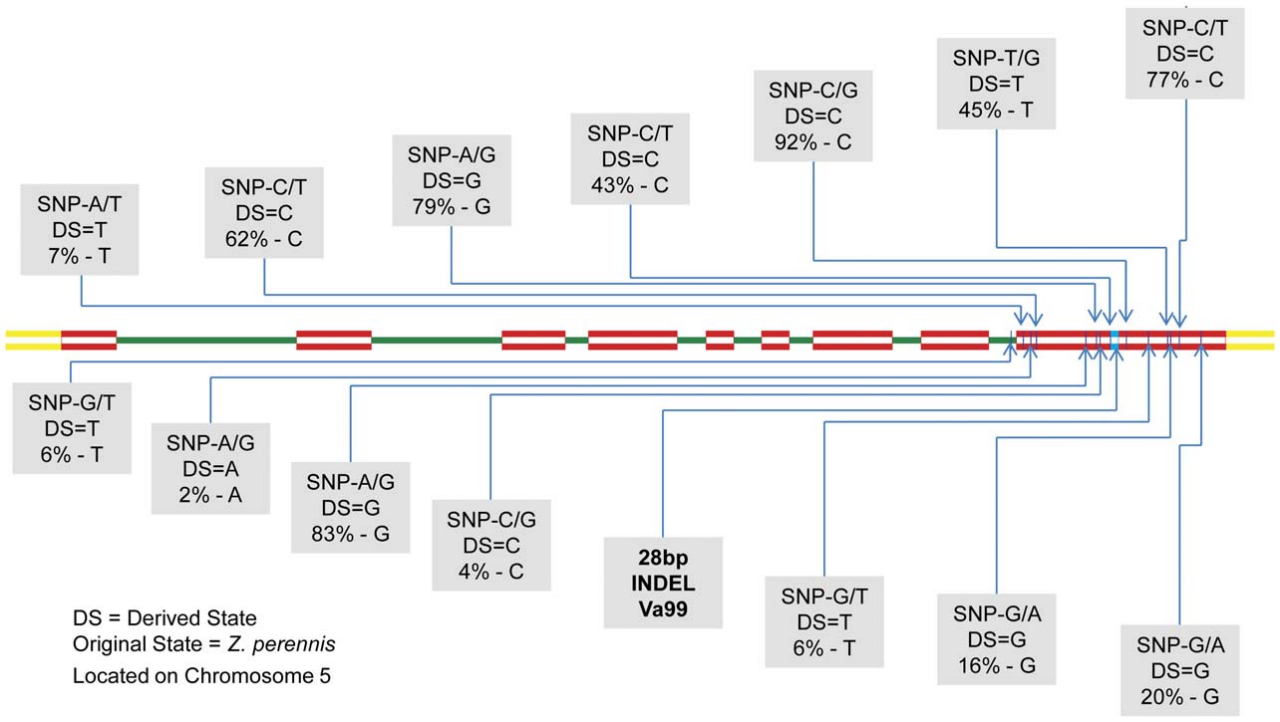
Sequence architecture of *ZmLOX5*. *ZmLOX5* consists of 9 exons (shown in red), and 8 introns (shown in green). *ZmLOX5* has a shorter second intron, spanning 511 bp. *ZmLOX5* is located on the reverse strand of chromosome 5:12,274,159–12,279,067, spanning 4908 bp (B73 RefGen_V2). Vertical blue lines in the final 3′ exon of the gene are the relative locations of SNPs discovered from Sanger sequencing. An InDel of 28 bp was found in the inbred line Va99 and located in the final exon of *ZmLOX5*, shown here in light blue. Their base pair change, derived state (as compared to *Z. perennis*), and derived state percentages are shown in the grey boxes.

Both *ZmLOX4* and *ZmLOX5* have the same genic architecture consisting of 9 exons and 8 introns. The ninth and final exon of both genes is the largest, which contains the conserved regions required for enzyme activity, and, due to its proximity to less conserved 3′untranslated region (UTR), is the only place where gene specific primers can be made [18]. PCR amplification of *ZmLOX4* and *ZmLOX5* specific products was difficult even on the small sample size of lines used for primer design and genotyping, and proved even more difficult when genetic diversity was increased across the lines included in the association panels. For *ZmLOX4*, of the 400 lines attempted, 20 did not amplify, 120 amplified but had sequence reads that were below quality thresholds set by the alignment software and 260 had useable sequence. For *ZmLOX5*, of the 400 lines attempted, 50 did not amplify, 147 amplified but had sequence reads that were below quality thresholds set by the alignment software and 203 lines had useable sequence. Sequencing and alignment of *ZmLOX4* and *ZmLOX5* sequences to the B73_RefGen_v2 revealed 9 SNPs in *ZmLOX4* (Figure 1) and 14 SNPs in *ZmLOX5* (Figure 2). A 5 bp long insertion was found in *ZmLOX4* in the 3′UTR and thus does not encode for any amino acid change. *ZmLOX5*, however, was found to have a 28 bp insertion in the ninth exon of inbred Va99 that results in a shift of the open reading frame. This would in turn mistranslate the highly conserved C-terminus of the enzyme and thus make the *ZmLOX5* allele in Va99 nonfunctional. Nucleotide diversity (π/bp) was 0.00054 for *ZmLOX4* and 0.0053 for *ZmLOX5*. Tajima's D test for neutrality values were −1.182 and −1.323 for *ZmLOX4* and *ZmLOX5*, respectively. Both values for Tajima's D are not significantly different from zero.

*Z. perennis* (Accession: 9475JAL88 Piedra Ancha) a perennial tetraploid teosinte considered to be a relative of ancestral maize [29] was sequenced at the *ZmLOX4* and *ZmLOX5* loci to establish the ancestral/derived state of the alleles in question. Derived state percentages varied between 0.3% and 97% in the maize panel and are reported on Figures 1 and 2; also, Table 1 outlines all SNPs found in the inbred lines, their location based on B73_RefGen_v2 and the percentage of the derived state that is seen in the inbred lines screened.

**Table 1:** SNP locations per B73 RefGen_v2 and their derived state percentages.

SNP Location	Base Pair Change	Derived State	Derived State %
***ZmLOX4***			
1:264224287	T/C	C	97
1:264224380	A/G	G	72
1:264224403	A/G	A	6
1:264224414	A/G/T	A/T	72/13
1:264224594	C/T	T	10
1:264224645	C/T	T	0.3
1:264224831	C/T	T	5
1:264224915	C/G	C	0.3
1:264224941	+/−	+	1
1:264224950	C/T	T	78
***ZmLOX5***			
5:12289458	G/T	T	6
5:12289504	A/T	T	7
5:12289534	A/G	A	2
5:12289555	C/T	C	62
5:12289748	A/G	G	83
5:12289789	A/G	G	79
5:12289804	C/G	C	4
5:12289845	C/T	C	43
5:12289877	C/G	C	92
5:12289963	G/T	T	6
5:12290038	T/G	T	45
5:12290050	G/A	G	16
5:12290083	C/T	C	77
5:12290167	G/A	G	20

### Presence/absence/duplication of *ZmLOX5*

**Figure 3:**
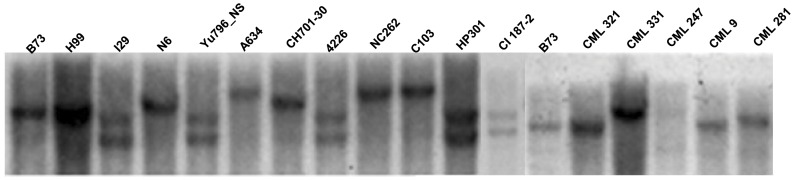
Southern blot of lines that did not PCR amplify for *ZmLOX5*. B73, confirmed to have a functioning ZmLOX5, is used as a control for the Southern blot. Five of the inbred lines screened show double banding: Yu796_NS, 4226, I29, HP301 and CI 187-2 and are suspected to have two copies of *ZmLOX5*. One is also missing a band: CML 247, and is suspected to have no copy of *ZmLOX5*.

**Figure 4:**
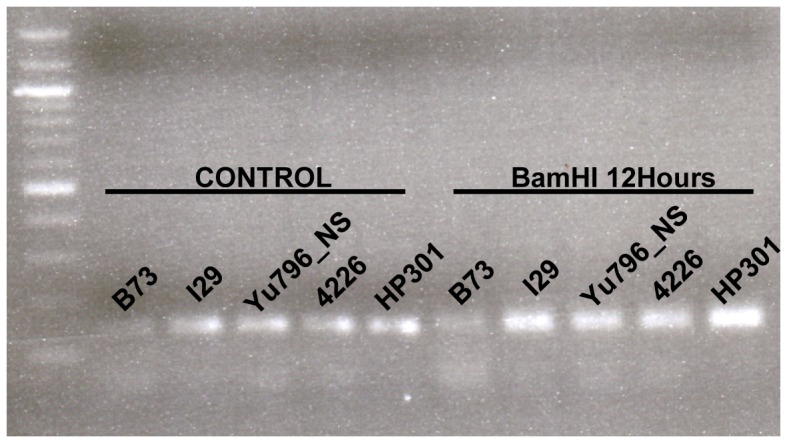
BamHI cut of *ZmLOX5* probe to check for a cut site within the probe. To rule out a BamHI cut site in the *ZmLOX5* probe used for Southern blotting, the probe was PCR amplified and then cut with BamHI. Shown here is an agarose gel with the untreated control (left) and BamHI treated (right) samples of four of the lines that showed two bands in the Southern blot. There is only one band in the BamHI treated lanes, showing that there is no cut site within the probe.

Of the 400 lines tested, 50 failed to amplify and were never sequenced for *ZmLOX5*. To better understand difficulties in amplification, we tested for presence/absence of *ZmLOX5* in these 50 lines using Southern blotting. Blot images (Figure 3) revealed that of the 50 lines tested, one (CML 247 PI595541) has not displayed any hybridization signal binding to the *ZmLOX5* gene-specific probe, suggesting that this line lacks *ZmLOX5*. Five of the lines screened (I29 Ames27115, Yu796_NS Ames27196, 4226 NSL30904, HP301 PI587131, CI 187-2 Ames26138) have two bands that strongly hybridized to the *ZmLOX5* probe, and the other 44 appear to be single copies. To rule out that there is a restriction enzyme site within the sequence hybridizing to the *ZmLOX5* probe, the probe region was PCR amplified and digested with BamHI restriction enzyme used for all the Southern blots presented. As shown in Figure 4, there is only one band in all samples, indicating that the four lines that showed two bands on the blot indeed contain two copies of *ZmLOX5*.

### LD and diversity in *ZmLOX4*/*ZmLOX5*

**Figure 5:**
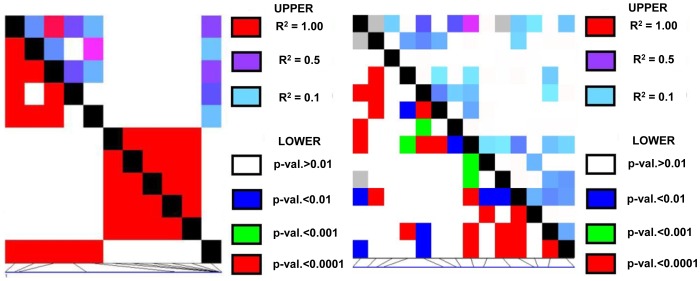
LD patterns in the C-terminus exon of *ZmLOX4* (left) and *ZmLOX5* (right). LD plots of SNPs found via Sanger sequencing (outlined in Figures 1 and 2, and Table 1) based on *ZmLOX4* with 6 SNPs and 1 InDel, spanning a total of 663 bp in the final 3′ exon across 260 diverse inbred lines, and *ZmLOX5* with 14 SNPs, spanning 709 bp across 203 diverse inbred lines. Both show a rapid (<100 bp) decay of LD (r^2^>0.1).

LD patterns in *ZmLOX4* and *ZmLOX5* were investigated in both sequence data that were collected in this experiment, and publically available data that is part of the Panzea project's HapMap genotypes search (available at www.panzea.org). Our sequencing data demonstrated that there was incomplete LD across the final exon of these genes (Figure 5). As seen in Figure 5, of the 9 SNPs and one InDel that were described earlier for *ZmLOX4*, only 6 SNPs (those above 10% frequency) and the InDel were considered, spanning a total of 663 bp across 260 lines. Each base pair of the InDel was included across the 5 bp that it spans. The InDel shows complete LD with itself, but not with any other polymorphism. Therefore, the final exon of *ZmLOX4* is not in complete LD, and LD decays rapidly (<100 bp) in this region despite some linkage being present. *ZmLOX5* shows a similar pattern of LD for the 14 SNPs considered (those above 10% frequency) across 203 lines and 709 bp. Again LD decays inside the final exon of *ZmLOX5*, similar to *ZmLOX4*.

**Figure 6:**
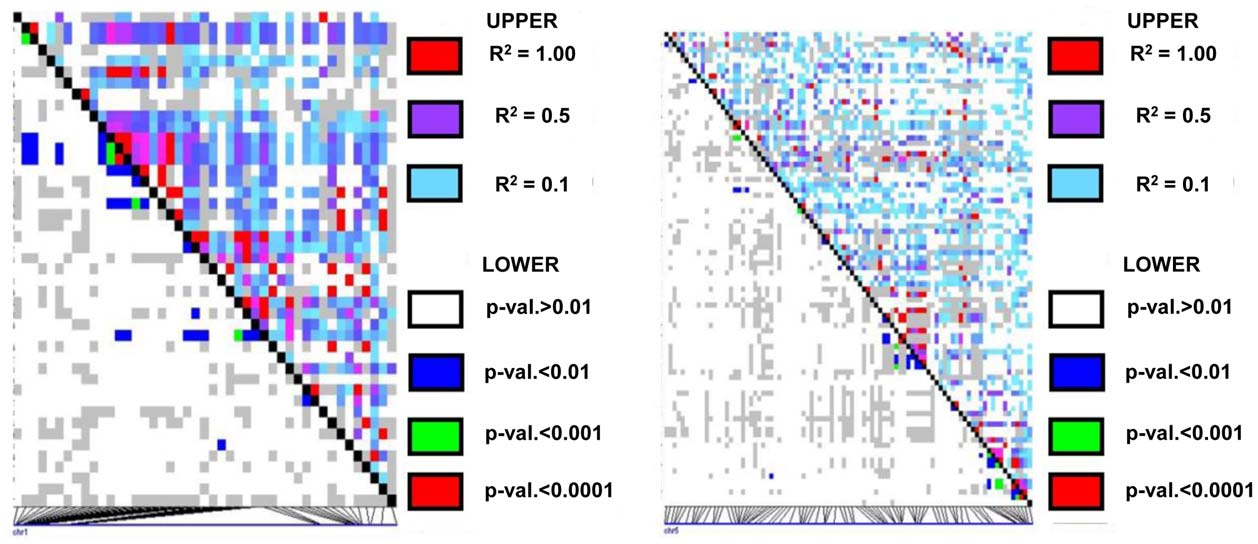
LD patterns across the whole locus of *ZmLOX4* (left) and *ZmLOX5* (right). LD plots of SNPs across the entire gene from the Maize HapMap in *ZmLOX4* (left) and *ZmLOX5* (right) Were based on 45 SNPs in *ZmLOX4* spanning 15,756 bp across the 27 NAM parents, and 91 SNPs in *ZmLOX5*, spanning 4884 bp across the 27 NAM parents. Again, we see a rapid (<100 bp) decay of LD (r^2^>0.1) though some moderate LD spans 250–300 bp.

The Maize HapMap data revealed 45 SNPs in *ZmLOX4* and 91 SNPs in *ZmLOX5* in the 27 lines sequenced. LD patterns across the entire locus of *ZmLOX4* and *ZmLOX5* reveal the same general decay pattern as our investigation of the active site. LD decays rapidly as seen by a lack of any relatively large linkage blocks (with an r^2^>0.1) in either of the LD plots (Figure 6), but small regions of a more moderate LD 250–300 bp are present. There is a difference in the LD structure of the two paralogs with *ZmLOX4* showing more extensive LD than *ZmLOX5*.

### LD at other members of the *ZmLOX* family

**Figure 7:**
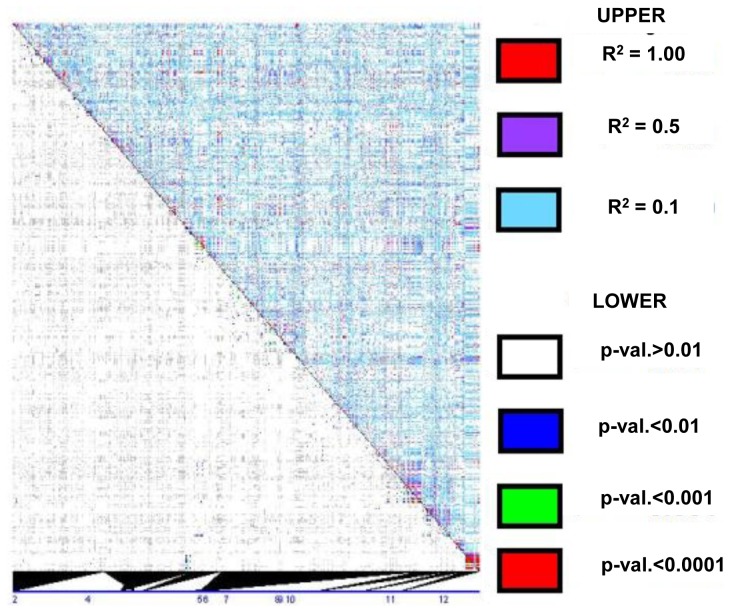
LD plot containing 10 members of the *ZmLOX* gene family. The LD plot of all SNPs from the Maize HapMap with 10 members of the *ZmLOX* gene family (*ZmLOX2, ZmLOX4, ZmLOX5, ZmLOX6, ZmLOX7, ZmLOX8, ZmLOX9, ZmLOX10, ZmLOX11, and ZmLOX12*) ordered numerically from left to right shows little LD across all of the *ZmLOX*s except for *ZmLOX12*, which is located in the lower right-hand corner.

**Figure 8:**
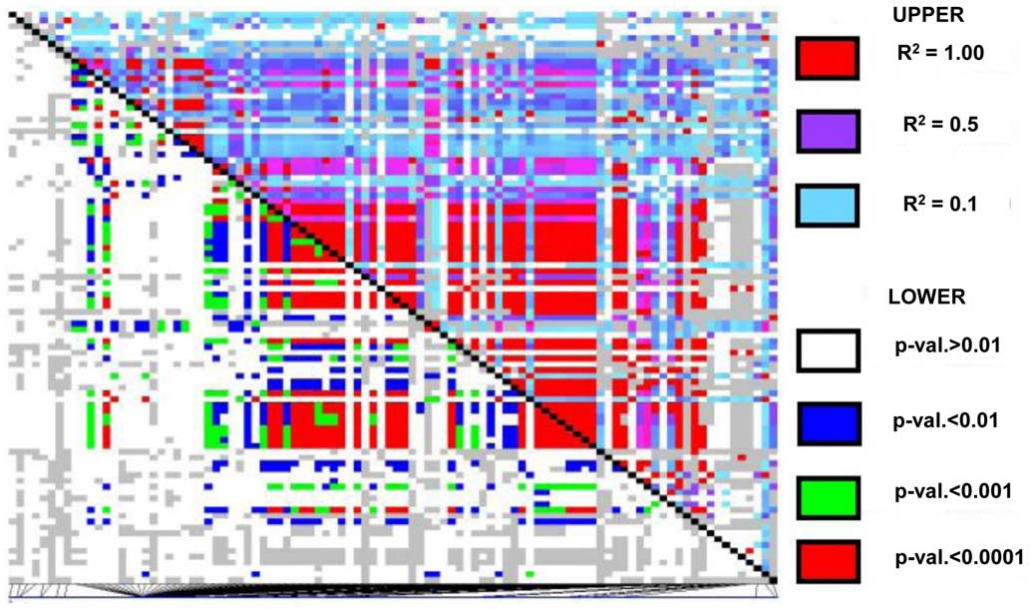
LD plot containing *ZmLOX12* and the 100 kb flanking the gene both upstream and downstream. The LD plot of SNPs from the Maize HapMap *ZmLOX12*, and the flaking 100 kb on either side of the gene shows that LD at this locus is very strong and extends farther than expected in maize. Approximately 3,000 bp across the *ZmLOX12* gene and extends beyond to approximately 19,000 bp. Further investigation of this locus revealed that there is a predicted gene of unknown function located very near (<100 bp) to *ZmLOX12*.

After observing how quickly LD decays within *ZmLOX4* and *ZmLOX5*, we investigated LD patterns in the other members of the *ZmLOX* family. LD patterns in Maize HapMap data at *ZmLOX4* and *ZmLOX5* were indeed similar for all but one of the other *ZmLOX* genes (Figure 7). In contrast to the rest of the *ZmLOX* family, *ZmLOX12* shows highly significant and correlated LD across the entire locus (>3000 bp) and the flanking sequence of 100 kb on either side of ZmLOX12 (Figure 8) shows LD extending beyond the *ZmLOX12* gene to a length of approximately 19,000 bp.

## Discussion

### LD pattern interpretation

Decay patterns of LD present in members of the *ZmLOX* family (except for *ZmLOX12*) are lower than typical LD patterns reported for single genes in maize [24], [30]. It is important to note that the extent of LD depends greatly on the population being investigated as the effects of population substructure will be present. The region of the genome being considered, recombination rate, mating design and the marker technology used also affects the extent of LD making comparisons across studies difficult. In light of this, we investigated whether or not there were significant differences in LD and diversity measures between the southern adapted and temperate germplasm and found none.

Not surprisingly, our LD results did not fully agree with HapMap data in the region of the gene we sequenced. This could be caused by our increased sample size and diversity; alternatively it seems likely that the shorter reads obtained in next generation sequencing used by the HapMap project might be misplacing some sequence due to the high similarity of the two genes. Thus next generation sequencing would be unable to distinguish or align the two paralogs, and potentially other high homology LOX genes. This shows that, despite being a powerful tool, there are limitations to current next generation sequencing technology. In contrast, unique SNP's were detected with next generation sequencing in each gene that unique primers cannot be reliably designed for. These SNP detection methods applied to regions of the genome are complementary.

*ZmLOX12* has a comparatively long (∼19,000 bp) linkage block within domesticated material. While LD this extensive is uncommon in the maize genome, HapMap data has found evidence of regions in the maize genome with LD spanning thousands to millions of bp [26]. Further analysis of this locus using the B73 maize genome shows that there is another unknown (but putative protein coding) gene in close proximity to (<100 bp) *ZmLOX12*. While a conclusion cannot be made on which gene is being selected upon, there is clear evidence that selection pressure is acting on these loci creating two distinct haplotypes. Only one of these two haplotypes is found in the temperate material, while both haplotypes segregate in tropical material. Combined with abnormal LD patterns it suggests that this variant might be important for temperate adaptation and warrants further testing. Unlike some of the other *ZmLOX*s, *ZmLOX12* has no documented function, and has no close homolog in any dicot species sequenced suggesting that it is a monocot specific *ZmLOX*. Further investigations of this gene could prove to be a valuable future target for plant breeders.

### Genetic diversity at the *ZmLOX4* and *ZmLOX5* loci

Both biological evidence for the conservation of normal physiological functions, and the conserved sequence data presented here suggest heavy historical selection pressure for retention of these genes. Using the sequence data, other measures of genetic diversity were considered to further characterize the two loci of interest, including nucleotide diversity (a measure of the degree of polymorphism within a population) and Tajima's D test for neutrality. Nucleotide diversity (π/bp) measures have been shown to vary 16-fold and have been related to chromosome structure, LD, recombination, and the population being investigated [24], [31]; the value for *ZmLOX5* is near those commonly reported for maize, however, the value for *ZmLOX4* is among the lowest reported for maize [24], [32]. The minimal LD observed and the results of Tajima's D leads to the conclusion that the polymorphisms present in these two genes are selectively neutral and have experienced no detectable selection. Low frequency mutations suggest that selection pressure on these genes has been more recently reduced, and there is likely some functional redundancy in the biochemical pathways these genes are involved in. Functional redundancy is supported by survival of single or even double knockout mutant lines for these two genes, which function very much like their wild-type relatives in terms of their ability to grow and reproduce under normal field conditions (not shown) [18]. It is believed that since *ZmLOX4* and *ZmLOX5* are located on different chromosomes, yet are still highly similar in sequence, these two genes have evolved from an evolutionarily recent segmental duplication event [18]. While the protein encoded by these two genes carry the same biochemical function, their differential expression in diverse organs in the plant and differential inducibility by various stimuli is what makes the two genes unique [18].

### Presence/absence of *ZmLOX5* and its implications

For the remaining 44 of *ZmLOX5* which did not amplify it is likely that the gene specific primer used to amplify *ZmLOX5* had no binding site in the 3′UTR. Contig alignments of the lines which were successfully sequenced (not shown) displayed high polymorphism in the 3′UTR of the gene, which could explain the difficulty in amplifying *ZmLOX5* using the original primer pair. Discovering that some lines had multiple copies of *ZmLOX5* while another was missing *ZmLOX5* was interesting and unexpected; however, this is not an uncommon occurrence among maize lines. Non-collinearity, hemizygosity or presence-absence variation where genetic loci can be present in one line but not in another, has become a more common observation within elite maize inbred lines [33], [34] than was previously expected. Furthermore, it has been documented among elite maize lines that functional genes can have different numbers of copies across lines (copy number variation [34], [35]). This may be due to unequal crossover events, transposition, or other unknown phenomenon [36]. Presence-absence variation and copy number variation is suspected to be one cause of the large amount of phenotypic diversity seen across maize species [34]. However, what is unique is the finding that a gene that is so rigorously conserved is missing or duplicated within lines that are considered to be elite and have been used in breeding programs around the world. Interestingly, two of the five lines confirmed to have duplicated *ZmLOX5*'s are popcorns as defined by previous subpopulation groupings [27], and there are only nine popcorn lines out of 400 individuals tested. While interesting, this may simply be due to a genetic bottleneck within the popcorns.

### Implications for association mapping studies

Based on the results presented in this study, it will be difficult to use LD patterns to associate a marker mutation in *ZmLOX4* or *ZmLOX5* to the phenotype. Conversely, if statistical associations are found between a drought tolerant or aflatoxin resistant phenotype, with *ZmLOX4* or *ZmLOX5*, respectively, then it is very likely that the marker associated with the phenotype is the causal mutation itself (accounting for population sub-structure and relatedness).

This study is among the first to investigate genetic diversity at important gene paralogs *ZmLOX4* and *ZmLOX5*. Conclusions that are drawn from this study will be directly applicable to association mapping of the traits they are hypothesized to effect. Because of the low frequency of the mutations we believe most important, disrupted *ZmLOX5* (Va99) and missing *ZmLOX5* (CML 247), it will not be possible to formally test these in this panel by association mapping, thus linkage mapping populations must be developed for testing.

## Materials and Methods

### Germplasm used

*ZmLOX4* and *ZmLOX5* were sequenced in an association mapping panel consisting of 400 inbred lines. 300 of these lines were originally put together as an association panel adapted to the temperate mid-west U.S. [27], but were bred in diverse locations such as France, Iowa, Mexico, Minnesota, North Carolina and Texas. While there is plentiful information on these 300 lines [27], many do not do well in the Southern U.S. and Texas; additionally they would have been unlikely to be selected for traits such as aflatoxin or drought tolerance that *ZmLOX4* and *ZmLOX5* condition. Therefore an additional 100 lines were selected to be part of an aflatoxin screening association panel adapted to the Southern U.S. bred either at CIMMYT in Mexico or in the Southern U.S. such as Mississippi, Georgia, Texas, and/or part of the Germplasm Enhancement of Maize (GEM) project [37]. In total, these lines should represent the vast majority of diversity in elite domesticated maize, with only the rarest of alleles not included. Of these 400 lines selected, 260 were successfully sequenced for *ZmLOX4* (see Table S1 for details) and 203 were successfully sequenced for *ZmLOX5* (see Table S2 for details). No permit was required for the field studies which were conducted on the Texas A&M University Research Farm in College Station, TX which is not privately owned or protected in any way. This field study did not involve any endangered or protected species.

### DNA extraction/PCR/sequencing

For sequencing, genomic DNA was extracted from V2 (second leaf) stage seedlings of the maize inbred lines of the association panel using the protocol as described by Zhang et al. [38]. Since sequence homology of *ZmLOX4* and *ZmLOX5* is high, gene-specific reverse primers (GSPs) were designed in the 3′ UTR of both genes to avoid amplification of both genes simultaneously during PCR reactions. Forward and reverse primer sequences are shown in Table 2 as well as expected amplicon size. The 3′ ends of the gene, where active sites are located [18], were isolated via PCR and sequenced using primers from Table 2. PCR reactions were carried out using the commercially available Qiagen Taq PCR Core Kit using Qiagen recommended protocols (available at http://www.qiagen.com/products/pcr/taqsystem/taqpcrcore.aspx#Tabs=t2). PCR conditions were: 1) 95°C for 5 minutes, 2) 35 cycles of 95°C for 45 seconds, 58°C annealing temperature for both *ZmLOX4* and *ZmLOX5* primers for 1 minute, and 72°C for 2 minutes, 3) 72°C for 10 minutes. Sequencing and PCR purification was carried out by DeWalch Life Technologies (http://ls.dewalch.com/, Houston, TX) using the terminator dye method. Sequences were then aligned using Sequencher 4.8 (http://www.genecodes.com, Gene Codes Corporation) and trimmed using internal trim algorithm in Sequencher 4.8. Clean and complete reverse and forward sequences were combined into consensus sequences and then aligned for comparison. Availability of the whole maize genome (http://www.maizesequence.org/index.html) allowed for the use of reference sequence data. Reference sequence contigs from B73 RefGen_V2 were used as an anchor to align experimental sequences. Sequence data used in this project can be accessed through GenBank (www.ncbi.nlm.nih.gov/genbank) under accession numbers JX033121–JX033380 for *ZmLOX4* and JX033380–JX033583 for *ZmLOX5*. As a source for comparison, *Z. perennis*, (CIMMYT accession 9476JAL87) an ancestral species of modern maize was used to establish the ancestral state of the polymorphism. SNP data for other *ZmLOX* genes was acquired from the Panzea project's “HapMap Genotypes Search” (found at http://www.panzea.org/db/searches/webform/marker_search_blob). Known locations of the other *ZmLOX*s based on the B73 RefGen_V2 locations were input into the query and resulting SNP data was used for analysis. LD was calculated using TASSEL, freely available software from the Panzea project (www.panzea.org) [39]. Molecular genetic diversity parameters were calculated using the aligned Sanger sequence data trimmed to equal lengths and analyzed in DNA Sequence Polymorphism [40], freely available software (www.ub.edu/dnasp/). The two diversity parameters calculated were nucleotide diversity (π) which is the average number of nucleotide differences per site between two sequences [41] and Tajima's D which is a statistic used to test Neutral Theory and whether or not directional selection has affected the region [40], [42].

**Table 2:** Gene specific PCR primers and expected amplicon sizes.

	Forward primer	Reverse primer	B73 theoretical amplicon size
*ZmLOX4*	5′ – TGC CGG ACC AGT CAA GCC CCT AC – 3′	5′ – CAC ACA TGA CAA CAT TAT CCA GAC G – 3′	948 bp
*ZmLOX5*	5′ – GCG GTG ATC GAG CCG TTC GTA ATC – 3′	5′ – CAA GCG TGG ACT CCT CTC TC – 3′	1266 bp

### Southern blot analysis of *ZmLOX5*

2-week old seedlings of maize inbred lines were used for extraction of genomic DNA as described by Zhang et al. [38] and 10 µg genomic DNA of each inbred line was digested with a restriction enzyme, *BamH* I, for overnight at 37°C. Digested DNA was electrophoresed in a 1.0% agarose gel prepared with Tris-acetate, EDTA (TAE) buffer, then transferred with 0.025 M phosphate buffer (pH 6.5) to the nylon membrane (Magna Nylon Transfer Membrane, Osmonics Inc., Minnetonka, MN, USA). The membrane with transferred DNA was cross-linked by UV Stratalinker 2400 and then hybridized in ULTRAhyb hybridization buffer (Ambion, Austin, TX, USA) with *ZmLOX5*-specific probe which is a 149 bp-fragment of 3′ UTR of *ZmLOX5* [18]. The probes were labeled using Ready-To-Go DNA Labeled Beads (GE Healthcare UK Limited) with ^32^P-dCTP according to the manufacturers protocol. Blot membranes were exposed to an X-ray film (Kodak, Rochester, NY, U.S.A.) in cassettes at −80°C for 3–14 days depending on the signal strength.

### Restriction digestion of *ZmLOX5* PCR fragment

Southern Blot Analysis of *ZmLOX5* in inbred lines showed that 5 inbreds have two *ZmLOX5* bands while other inbred lines have single or no band, indicating either these inbreds have two *ZmLOX5* genes or *ZmLOX5* genes in these inbred lines have *BamH* I site in the probe region. We PCR amplified this region of *ZmLOX5* from these inbreds. Purified PCR products were digested with *BamH* I and the electrophoresis of the digestion to check if there is *BamH* I site in the probe region of *ZmLOX5* of these inbreds.

## Supporting Information

Table S1 **SNP genotypes for the 260 lines successfully sequenced for** ***ZmLOX4*** **and their respective position on chromosome 1 based on the B73 reference genome (B73 RefGen_V2).**
(XLSX)

Table S2 **SNP genotypes for the 203inbred lines successfully sequenced for** ***ZmLOX5*** **and their respective position on chromosome 5 based on the B73 reference genome (B73 RefGen_V2).**
(XLSX)
